# Hydrogel Capacitors Based on MoS_2_ Nanosheets and Applications in Glucose Monitoring

**DOI:** 10.3390/molecules29184401

**Published:** 2024-09-16

**Authors:** Yizhi Wang, Jinwen Zhang, Yusen Zhang, Bing Wang, Yang Zhang, Hui Lin

**Affiliations:** 1 School of Intelligent Science and Control Engineering, Jinling Institute of Technology, Nanjing 211169, China; w_yz@jit.edu.cn (Y.W.); w1369014210@163.com (Y.Z.); 15256529815@163.com (B.W.); 2 College of Material and Chemical Engineering, Minjiang University, No. 200, Xiyuangong Road, Minhou District, Fuzhou 350108, China; y0087@mju.edu.cn; 3 School of New Energy and Materials, Ningde Normal University, No. 1, Xueyuan Road, Jiaocheng District, Ningde 352100, China

**Keywords:** MoS_2_ nanosheets, dielectric property, microneedle technology, interstitial fluid glucose monitoring

## Abstract

Non-invasive/minimally invasive continuous monitoring of blood glucose and blood glucose administration have a high impact on chronic disease management in diabetic patients, but the existing technology is yet to achieve the above two purposes at the same time. Therefore, this study proposes a microfluidic microneedle patch based on 3D printing technology and an integrated control system design for blood glucose measurement, and a drug delivery control circuit based on a 555 chip. The proposed method provides an improved preparation of a PVA-PEG-MoS_2_ nanosheet hydrogel, making use of its dielectric properties to fabricate a microcapacitor and then embedding it in a microfluidic chip. When MoS_2_ nanosheets react with interstitial liquid glucose (and during the calibration process), the permittivity of the hydrogel is changed, resulting in changes in the capacitance of the capacitor. By converting the capacitance change into the square-wave period change in the output of the 555 chip with the control circuit design accordingly, the minimally invasive continuous measurement of blood glucose and the controlled release of hypoglycemic drugs are realized. In this study, the cross-linking structure of MoS_2_ nanosheets in hydrogel was examined using infrared spectroscopy and scanning electron microscopy (SEM) methods. Moreover, the critical doping mass fraction of MoS_2_ nanosheets was determined to be 2% via the measurement of the dielectric constant. Meanwhile, the circuit design and the relationship between the pulse cycle and glucose concentration is validated. The results show that, compared with capacitors in series, the microcapacitors embedded in microfluidic channels can be connected in parallel to obtain better linearized blood glucose measurement results.

## 1. Introduction

In the past decade, diabetes has become one of the most vital threats to human health with its soaring prevalence. For diabetic patients, both persistently high blood glucose levels due to lack of management and excessively low blood glucose levels from over-management cause irreversible damage to the human body and even trigger severe complications that endanger patients’ lives. Therefore, it is crucial for diabetic patients to have their blood glucose levels continuously and steadily controlled within the prescribed range, and continuous and accurate monitoring of blood glucose is the first step toward achieving this stable control [1]. At present, depending on different sampling times and data collection principles, common methods for monitoring blood glucose can be divided into invasive/non-invasive/minimally invasive monitoring and intermittent/continuous monitoring. The most common, fingertip blood collection and various portable glucose meters, are invasive and intermittent, typically using colorimetric methods [2,3] to determine glucose concentration, which are accurate, and renowned for their price, but possibly lead to infections and lack real-time monitoring capability. Other methods include electrochemical methods [4], fluorescence methods [5], and near-infrared spectroscopy methods [6]. Emerging methods include electrocardiograph (ECG) signal analysis [7] and continuous glucose monitoring (CGM) [8], which capture real-time blood glucose concentrations through the data generated when the interstitial fluid in the body reacts electrochemically with the implanted sensors. This method reduces the pain of daily blood collection for patients; however, the data signals captured are too weak to drive the control system for online drug delivery.

Achieving high adherence to proactive glycemic control in diabetic patients remains a central concern in chronic disease management. The methods for improving patients’ adherence mainly involve reducing the pain associated with blood glucose monitoring and the administration of glucose-lowering drugs. Apart from the common methods, 3D printing-based hollow microneedles become prevalent due to their ability to extract body fluids and facilitate transdermal drug delivery tailored to individual patients’ needs [9]. However, as far as calibration methods for blood glucose monitoring are concerned, most of them rely on principles like photothermal [10] and colorimetric methods [11] and reduction current measurements [12]. The outputs of the first two principles are fluorescence intensity and grayscale values. These outputs, however, can hardly be used to drive drug delivery control systems. In addition, reduction current measurements are typically employed for intermittent monitoring and cannot achieve the function of drug delivery control based on blood glucose concentration. Moreover, some studies have implemented iontophoresis-driven drug delivery systems that can automatically administer drugs based on blood glucose concentration. Nevertheless, the entire drug delivery process lacks visualization and data feedback, making it impossible to control the drug delivery process. Patients are unaware of the use of medication fluid, which poses certain risks. To this end, when microneedle technology is applied to the fields of blood glucose monitoring and drug delivery control, there is an urgent need for a device that can continuously generate signals based on blood glucose concentration. These signals should be able to drive the drug delivery system, thereby expanding the application scope of microneedles.

Regarding the emerging new material for flexible sensors, molybdenum disulfide (MoS_2_) crystals and their copolymers have been widely used as important solid lubricants and lubricant additives for mechanical protection across various devices since the mid-19th century due to their excellent friction properties, specifically reducing friction at low temperatures and increasing friction at high temperatures, thereby effectively extending the service life of devices. With the rise of nanomaterials and improved preparation techniques, nanocompounds of MoS_2_ and polymer materials were synthesized in 1994 by Benavente et al. [13], while MoS_2_ nanosheets were prepared via lithium intercalation and exfoliation for the first time in 2002 by Lemmon et al. [14]. Thereafter, monolayer or few-layer MoS_2_ nanosheets have exhibited semiconductor properties [15], optoelectronic device characteristics [16], piezoelectric properties [17], and enzyme-like properties [18], among others, soon establishing themselves as highly-sought-after materials. Particularly, the significant electrocatalytic activity of MoS_2_ nanosheets, akin to that of catalase (CAT) and glucose oxidase (GOx), has propelled their widespread adoption in various applications, including biosensors and electrochemical sensors in recent years. These sensors convert the concentrations of the target into quantitative signals using different principles. For example, Lin et al. [19] achieved the calibration of H_2_O_2_ concentration using colorimetric methods. At present, one of the mainstream research directions is enzyme-based nanocomposites, whose calibration principle is to immobilize enzymes on bio-probes made from MoS_2_ and its composites. This approach utilizes the current generated from the redox reaction between the enzyme on the probe and the target substance to quantitatively analyze the concentration of the target substance. Typical representatives in this regard include Au–MoS_2_ glucose probes [20], Pt–MoS_2_-cascaded nano-reactors [21], MoS_2_ quantum dot (QD)/Pd@rGO composite materials for detecting the drug nevirapine (NVP) [22], and MoS_2_ thin film/xanthurenic acid (Xa) composite materials for calibrating purine substances [23]. Some studies have attempted to use MoS_2_ nanosheets based on non-enzymatic methods to calibrate glucose and hydrogen peroxide [24,25]. However, the resulting electrical signals are weak and need to be re-amplified before they can be used for the online release of glucose-controlling drugs.

From the perspectives of components measured and circuit design, apart from the applications for the detection of displacement [26] or replacement of oil and liquid [27], the dielectric properties of capacitive sensors are also widely employed for continuous and online measurements of the changes in grain moisture [28,29]. These sensors offer advantages such as a simple structure, high resolution, strong anti-interference capability, and rapid dynamic response. Since the object of monitoring is moisture, they lack specificity, and the monitoring results are easily affected by multiple interferences. Additionally, Huang [30] designed a chaotic circuit based on a 555 chip using the principle of chaotic measurement and applied it to non-invasive blood glucose monitoring based on the metabolic heat integration method. They also established a mathematical model through polynomial fitting, but did not carry out research on the non-invasive online monitoring of blood glucose levels.

Based on the analysis above, this study introduces an innovative solution for the online monitoring and control of blood glucose levels, applicable to microneedles, based on several aspects mentioned above. To begin with, MoS_2_ nanosheets were doped in a PVA-PEG solution, and the PVA-PEG-MoS_2_ mixture was dried to prepare a colloid. The cross-linking structure in the PVA-PEG-MoS_2_ colloid was then determined using infrared spectroscopy and scanning electron microscopy (SEM). How the dielectric constants of the colloids varied with different mass fractions of MoS_2_ nanosheets was further investigated. Moreover, this study also presents a novel method for the continuous online monitoring of interstitial fluid glucose and introduces an integrated microneedle structure that can be utilized to control drug delivery. Using the specificity with which MoS_2_ nanosheets were consumed during the non-enzymatic calibration of glucose, we fabricated microcapacitors, and then embedded them in a parallel manner within a microfluidic chip. When the interstitial fluid extracted passed through the microfluidic chip, the dielectric constant of the PVA-PEG-MoS_2_ gel dielectric changed, which, in turn, caused the total capacitance value to change. Then, a 555 timer chip was employed to design a circuit and convert capacitance changes into variations in output signal periods, which ultimately achieved the online measurement of blood glucose. This signal can also be used to control the release of glucose-controlling drugs. Finally, the feasibility of the proposed method was validated using Multisim circuit design and a MATLAB simulation analysis.

## 2. Results

### 2.1. Infrared Spectroscopy Results and Analysis

**Figure 1:**
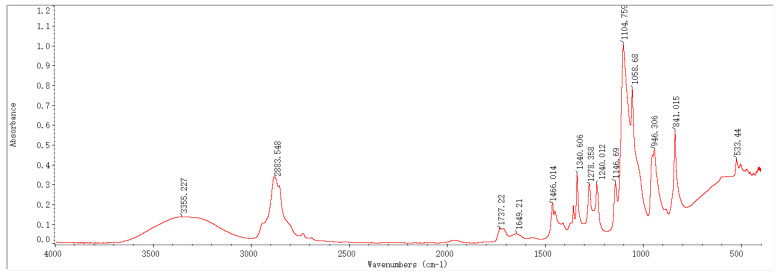
FTIR spectrum of a PEG-PVA sample (control group: MoS_2_: 0%).

**Figure 2:**
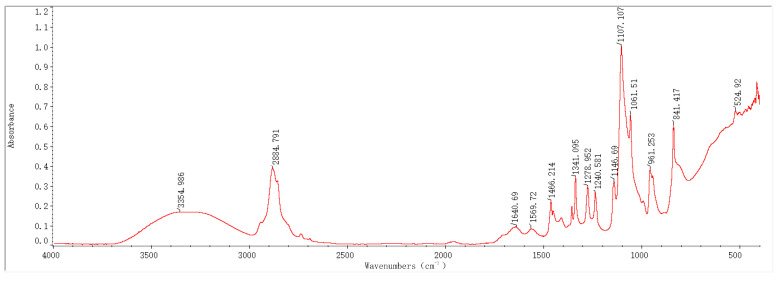
FTIR spectrum of a PEG-PVA-MoS_2_ sample (group 1: MoS_2_: 0.8%).

**Figure 3:**
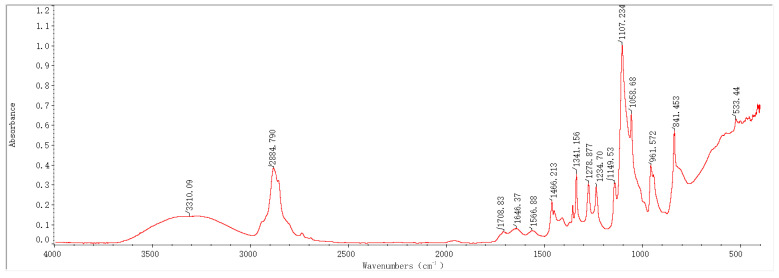
FTIR spectrum of a PEG-PVA-MoS_2_ sample (group 2: MoS_2_: 1.2%).

**Figure 4:**
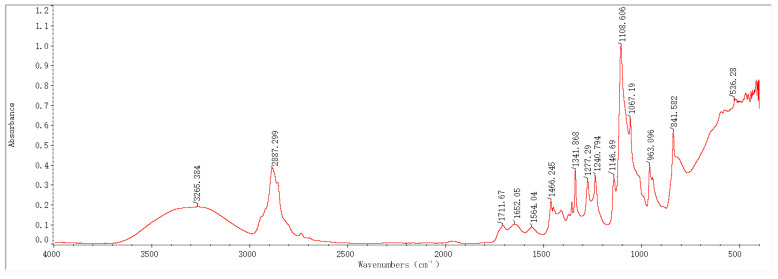
FTIR spectrum of a PEG-PVA-MoS_2_ sample (group 3: MoS_2_: 2%).

In recent years, Fourier-transform infrared spectroscopy (FTIR) has been widely applied as a key technique to monitor the composition of organic/inorganic functional groups. In this study, based on FTIR technology, the infrared absorption peaks of samples before and after the preparation of PEG-PVA-MoS_2_ hydrogels were analyzed, with a view toward exploring changes in their functional group composition and structure. The characteristic peaks in the FTIR spectra of the hydrogel samples prepared with different MoS_2_ mass fractions were annotated and analyzed, as shown in Figure 1, Figure 2, Figure 3 and Figure 4.

**Figure 5:**
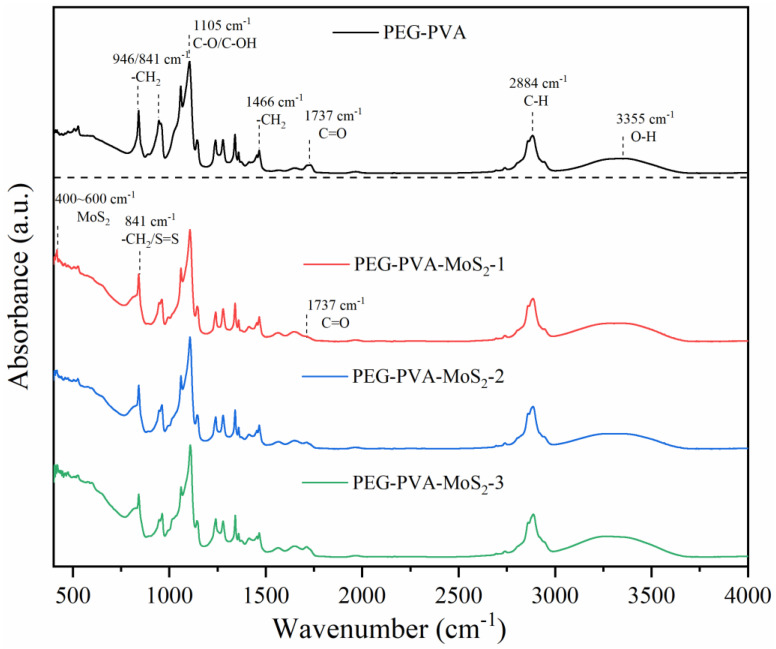
FTIR spectrum features of the PEG-PVA-MoS_2_ hydrogel for the four groups.

The FTIR spectrum features of the PEG-PVA-MoS_2_ hydrogel before and after preparation are shown in Figure 5. For the PEG-PVA sample, the absorption peak at 3355 cm^−1^ was attributed to the O–H stretching vibration mode, exhibiting a broad peak shape due to varying degrees of hydrogen bonding. The overlapping peak at 2884 cm^−1^ was attributed to the C–H stretching vibration modes of PEG and PVA. The peak at 1737 cm^−1^ was related to the C=O stretching vibration mode of the ester group in PVA. The peak near 1466 cm^−1^ was attributed to the bending vibration mode of methylene in PEG. The absorption peaks at 1341 cm^−1^, 1278 cm^−1^, and 1240 cm^−1^ originated from the deformation vibration or out-of-plane wagging vibration of methylene in PEG. The overlapping peaks observed within the range of 1147–1059 cm^−1^ were attributed to the C–O and C–OH (terminal hydroxyl) stretching vibration modes of PEG. The peaks at 946 cm^−1^ and 841 cm^−1^ were related to the in-plane rocking vibration mode of methylene in PEG molecules. These characteristic peaks were simple overlays of spectra of PEG and PVA, indicating the formation of hydrogen bonds between the hydroxyl groups of PEG and the hydroxyl groups of PVA molecular chains, anchoring PEG to PVA chains and cross-linking them through primarily physical interactions.

### 2.2. SEM Analysis

**Figure 6:**
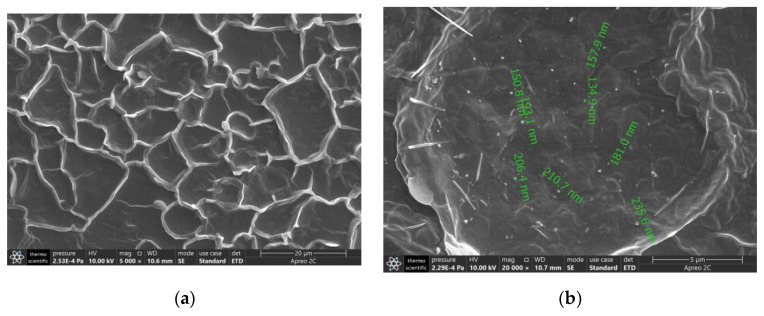
SEM results: (**a**) cross-linking reaction of PVA-PEG (×5000 magnification); (**b**) image of PVA-PEG-MoS_2_ (×20,000 magnification).

The SEM results as in Figure 6a,b clearly show that the cross-linking product formed by PVA-PEG encapsulates the MoS_2_ nanosheets, which prevented them from oxidizing and losing their original efficacy due to air and temperature conditions.

### 2.3. Dielectric Constant Testing

**Figure 7:**
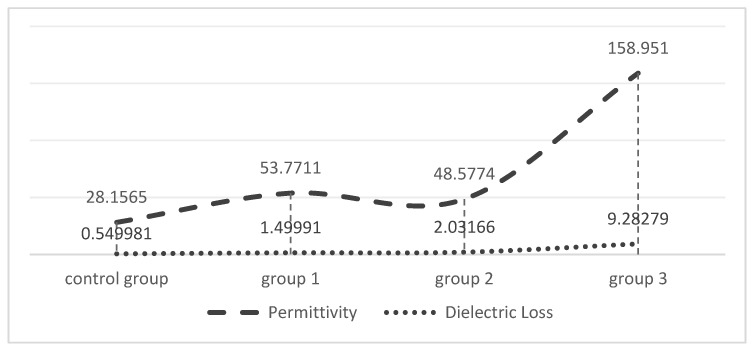
Analyses of dielectric constant and dielectric loss.

The dielectric constants of the prepared hydrogel were measured and analyzed as shown in Figure 7. In this project, the measured samples included the following experimental groups: group 1 (0.8% MoS_2_), group 2 (1.2% MoS_2_), group 3 (2% MoS_2_), and the control group (0% MoS_2_). The experimental results show that, under the same measurement frequency of 1000 Hz, the hydrogels prepared with different experimental setups demonstrate significant differences in dielectric constants. As illustrated in Table 1, initially, the dielectric constant of the hydrogels shows a slight decrease as the doping concentration of MoS_2_ increases, followed by a sharp increase. The dielectric loss also exhibited an upward trend with higher concentrations of MoS_2_ doping, particularly exceeding 2%. Additionally, it was concluded that the dielectric constant and loss values of the hydrogel were lower in the control group than in the other experimental groups.

### 2.4. Relationship between Real-Time Blood Glucose Concentration and the Output Period of the 555 Chip

#### 2.4.1. Capacitors in Series

**Figure 8:**
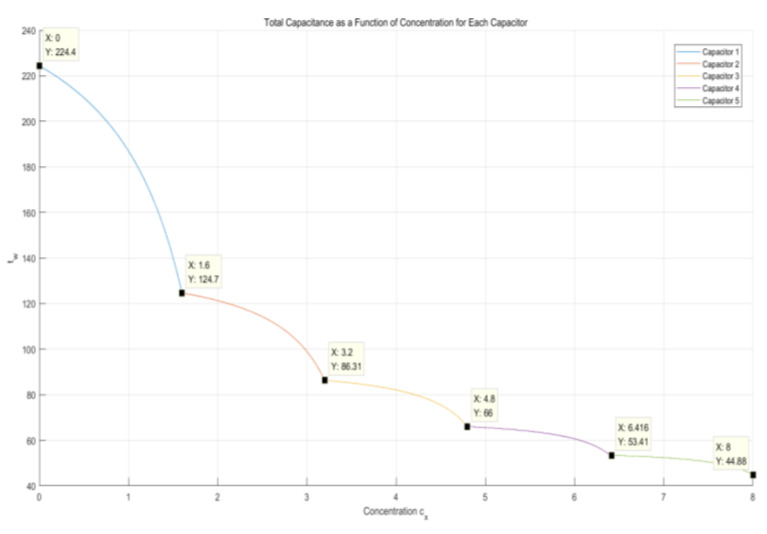
Changes in total capacitance in capacitors connected in series.

Suppose that five capacitors are connected in series on the microfluidic chip, each with an initial value of 10 mF and a minimum value of 1 mF after the consumption of the capacitor. The overall circuit equivalent resistance of the 555 chip is 510 kΩ. At the body surface temperature, the glucose in the interstitial fluid consumes MoS_2_ nanosheets in a fixed proportion of μ, leading to an increase in the total capacitance in series on the microfluidic chip, thereby affecting the output clock period of the 555 chip. This process was simulated using MATLAB, and the results are illustrated in Figure 8.

#### 2.4.2. Capacitors in Parallel

**Figure 9:**
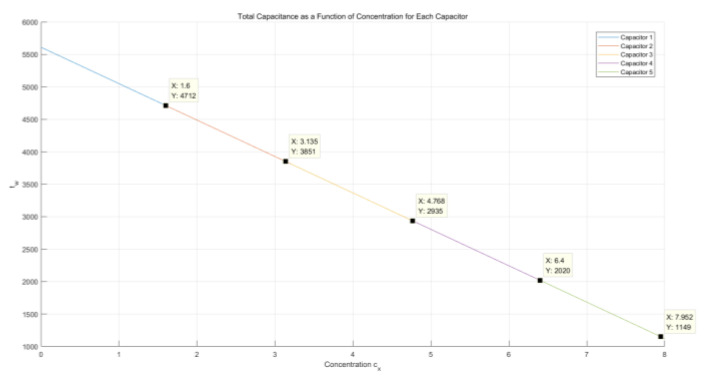
Changes in total capacitance in capacitors connected in parallel.

Suppose that five capacitors are embedded and connected in parallel in the microfluidic channels, each with an initial value of 10 mF and a minimum value of 1 mF after the consumption of the capacitor. The overall circuit equivalent resistance of the 555 chip is 510 kΩ. At the body surface temperature, the glucose in the interstitial fluid consumes MoS_2_ nanosheets in a fixed proportion of μ (for computational simplicity, here, μ is set to 1), leading to a decrease in the total capacitance in parallel on the microfluidic chip, as shown in Figure 9.

It is important to note that, due to different measurement methods and operating temperatures, the consumption of MoS_2_ nanosheets by glucose does not always follow a proportional relationship with μ. In specific cases, μ can be determined through calibration, but the changes in μ do not affect the overall trend of capacitance changes described above.

#### 2.4.3. The Output Period of the 555 Chip

**Figure 10:**
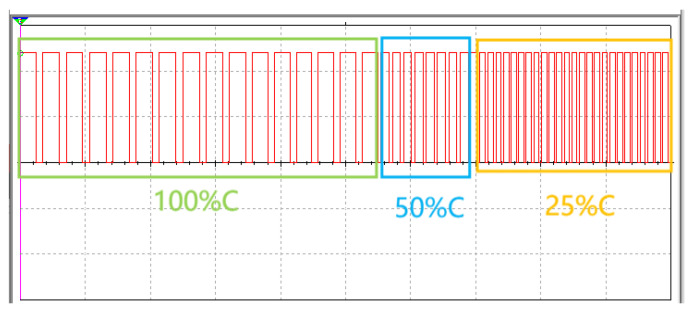
Relationship between capacitance changes and output signal periods of the 555 chip.

Whether capacitors are embedded in series or in parallel in the microfluidic chip, they are connected in series in the designed 555 chip circuit in the form of total capacitance. As long as the dielectric constant of the capacitors is changed, the output clock period of the 555 timer chip is affected. The specific change relationship is illustrated in Figure 10.

## 3. Discussion

### 3.1. Physical Structure Analysis of the PVA-PEG-MoS_2_ Hydrogel

With the doping of MoS_2_, the sample spectra exhibited significant changes. The relative intensity of absorption peaks within the range of 400–600 cm^−1^ notably increased, which is primarily related to the lattice vibration mode of MoS_2_. The shape of the absorption peaks in this range depends on the particle size. When atoms accounted for a larger percentage of the surface, it resulted in an increase in the intensity of absorption peaks related to the longitudinal phonon vibration and stretching. It was presumed that MoS_2_ had been uniformly doped on the surface structure of the PEG-PVA hydrogels, which can be further analyzed with the cloud TEM results. The absorption peak at 841 cm^−1^ also stretched significantly, which is mainly influenced by the S=S stretching vibration in MoS_2_, a critical indicator of the successful cross-linking of MoS_2_. Additionally, a red shift was observed in the C=O absorption peak at 1377 cm^−1^. Typically, the lower the wavenumber of a C=O stretching vibration, the stronger the hydrogen bonding system. Thus, the doping of MoS_2_ probably altered the hydrogen bonding network of PEG-PVA. Generally, the absorption peaks within the range of 3000–3600 cm^−1^ can be used to measure the change pattern of the hydrogen bonding system of samples. The fitting results show that the PEG-PVA sample exhibits three distinct sub-peaks within the range of 3000–3600 cm^−1^. The absorption peak near 3239 cm^−1^ was mainly attributed to the O–H structure with strong hydrogen bonding coordination approaching a valence of 4. The absorption peak near 3387 cm^−1^ primarily originated from O–H molecules with distorted hydrogen bonds, characterized by a weaker bonding strength. The absorption peak near 3497 cm^−1^ was mainly related to free O–H structures. With the doping of MoS_2_, the relative intensities of the absorption peaks near 3239 cm^−1^ and 3387 cm^−1^ gradually increased, while the free O–H structure gradually weakened, confirming that the doping of MoS_2_ led to the formation of stronger hydrogen bond interactions among molecules and enhanced the mechanical properties of the hydrogel samples. Additionally, with increasing the doping of MoS_2_, a new absorption peak appeared near 1740 cm^−1^, indicating bonding interactions between MoS_2_ and –H in free O–H, forming a C=O structure. For this reason, the doping of MoS_2_ resulted in a dual cross-linked structural system in both physical and chemical manners, significantly altering the physicochemical properties of the colloids.

In Figure 6a, the formation of cross-linking products is evident; the ring structures represent the cross-linking products of the flexible substrate after drying. The sheet-like structure shown in Figure 6b is the encapsulated MoS_2_ nanosheets. It can be seen that the encapsulant well encapsulated the MoS_2_ nanosheets. Combined with the SEM results, the analysis results of the FTIR spectrum in Figure 5 were validated, proving that MoS_2_ was uniformly doped on the surface structure of the PEG-PVA hydrogel.

### 3.2. Doping Concentrations of MoS_2_ and the Dielectric Constant

As shown in Figure 7, the doping concentration of MoS_2_ significantly influenced the dielectric constant and loss values of the hydrogel. The doping concentration of MoS_2_ exhibited a direct proportionality relationship with the dielectric constant of the prepared hydrogel. Specifically, with the increase in the mass fraction of the doped MoS_2_, the dielectric constant of the hydrogel also grew. Beyond a mass fraction of 2%, the dielectric constant of the prepared hydrogel rapidly rose, accompanied by an increase in the dielectric loss. Thus, it was observed that a mass fraction of 2% acted as a critical point: below this point, the changes in the MoS_2_ mass fraction yielded minimal alterations in the dielectric constant of the hydrogel, approximately regarded as 0; above this point, the dielectric constant of the prepared hydrogel became more sensitive to the changes in MoS_2_ mass fraction.

### 3.3. Parallel and Series Connections of Capacitors and Changes in the Output Periods of the 555 Chip

Figure 8 and Figure 9 illustrate the variations in MoS_2_ with the consumption of glucose reflected in the total capacitance when microcapacitors were embedded in the microfluidic chip in parallel and series, respectively. It was observed that, as the interstitial fluid flowed through the designed microcapacitors, MoS_2_ nanosheets and glucose in the interstitial fluid mutually consumed each other in certain ratios. The total capacitance connected in series in the circuit showed a downward trend because of the changes in the dielectric constants of the capacitors until the dielectric constants of all capacitors no longer varied. However, the different connection methods of the capacitors determined different downward trends of the total capacitance. Figure 9 demonstrates that, when microcapacitors are connected in parallel, the changes in the total capacitance in the microfluidic chip show better linearity. In contrast, Figure 8 illustrates that, when microcapacitors are connected in series, the changes in the total capacitance exhibit a nonlinear segmented quality, and the overall downward trend is not linear, but rather approaches the depletion of the capacitance value slowly and indefinitely. The total capacitance of the microfluidic chip was converted to the change in the output signal period of the 555 chip, as shown in Figure 10, when the capacitors were connected in series. In this case, the change in the output signal period was more challenging to detect, especially when the capacitance value approached depletion, which caused the change in total capacitance to become milder and smaller, resulting in a more limited change in the output signal period. Based on this consideration, the microcapacitors in the microfluidic chip should be connected in parallel.

## 4. Materials and Methods

### 4.1. Chemicals

(1) Experimental materials: Small-sized dispersion of monolayer/few-layer MoS_2_ nanosheets; 1 bottle, 500 mL (1 mg/mL) (purchased from Nanjing MKNANO Tech. Co., Ltd., Nanjing, China); self-made encapsulant (PVA-PEG 1:2); and laboratory distilled water, 500 mL.

### 4.2. Instruments

(1) Hydrogel preparation:

Experimental setup: 85-2 digital temperature-controlled magnetic stirrer(Shanghai Meiyingpu Instrument Manufacturing Co., Ltd, Shanghai, China); SF-400 electronic balance(FoshanPengchang Construction Machinery Co., Ltd, Foshan, China); XGQ-2000 electric thermostatic incubator (Suyida Equipment Co., Ltd, Wuxi, China); and BKMAM magnetic stirrer (B-type, 8 × 30 mm)(Bikemanbio co., Ltd, Changde, China).

Experimental process parameters: Mixing time of MoS_2_ nanosheets and embedded agent: 4 h (at room temperature); drying temperature: 40 °C.

(2) Infrared spectroscopy analysis:

Instrument model: Thermo Scientific Nicolet Summit X in Laboratory (Waltham, USA), Fourier transform type, with a signal-to-noise ratio of 30,000:1.

Testing method: The prepared samples were measured using an IRSpirit FTIR spectrometer(Thermo Fisher Scientific, Waltham, USA), with KBr as the background at room temperature. Each sample was scanned 32 times cumulatively for its FTIR signal, covering a frequency range of 4000–400 cm^−1^ and a resolution of 0.5 cm^−1^. The data were then processed using OMNIC v8.0 software for absorbance/transmissivity conversion, automatic smoothing, etc.

(3) SEM analysis:

Testing instrument: Thermo Scientific Apreo 2C (Thermo Fiser Scientific, Waltham, USA), with Oxford ULTIM MAX65 spectroscopy(Oxford Instruments, Oxford, UK), with metal spraying on sample surface.

(4) Dielectric constant test was conducted at room temperature, using Novocontrol Concept 80 (Novocontrol Technologies Gmbh&CO.KG, Montabaur, Germany).

(5) Circuit design and data analysis:

Circuit design and simulation were performed using Multisim 14.0; data analysis and simulation were performed using MATLAB R2017a.

### 4.3. Overall Methodology

**Figure 11:**
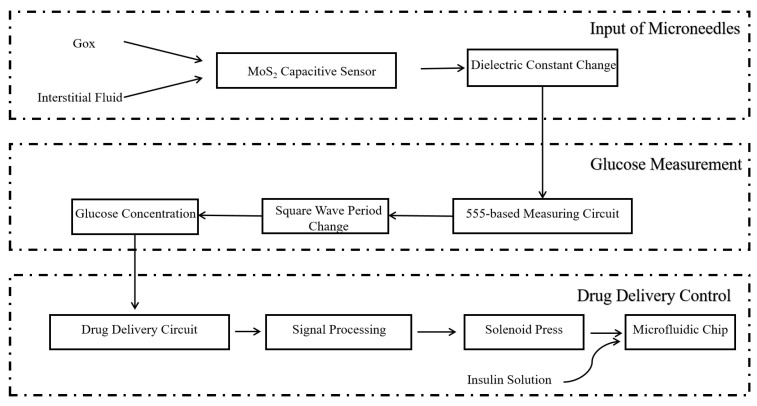
Overall design methodology.

**Figure 12:**
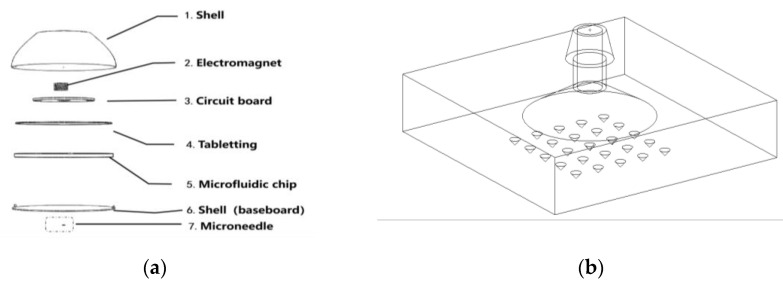
Schematic design of the microneedle patch: (**a**) overall structure of the microneedle patch; (**b**) schematic of the extraction of interstitial fluid based on 3D-printed microneedles.

Figure 11 illustrates the overall research plan for this project. Figure 12a depicts the overall structure of the designed microneedle patch, while Figure 12b provides detailed information on the microneedle design. The overall methodology follows the trace of chained changes: firstly, the consumption of MoS_2_ nanosheets cross-linked in the hydrogel in calibrating glucose will change the dielectric constant of hydrogel capacitors, followed by the change in the capacity of the capacitors. As the capacitance change will influence the output frequency of the 555 timing chip, a relevant design of the circuit is required. Therefore, the following critical procedures and validation methods were designed for the feasibility of the proposal:

(1) Regarding the feasibility of glucose calibration using the hydrogel capacitive sensors: The critical material, the PVA-PEG-MoS_2_ nanosheet hydrogel, for the capacitive sensors was prepared in accordance with the designed procedures, followed by the validation of the required dielectric constants to determine the sensitivity of the MoS_2_ mass fraction in the hydrogel for glucose calibration, to see if the relative dielectric constant would change as expected.

(2) Regarding signal transfer: With the satisfactory results of the capacitance change of the designed hydrogel after glucose calibration, the conversion mechanism where blood glucose concentration signals caused the capacitance to change was proposed and realized through a circuit design, which was used to calculate and derive the relationship between the capacitance change and blood glucose concentration. Meanwhile, the method of connecting capacitors in series or in parallel to achieve the purpose of this design were validated through simulations.

(3) Regarding the drug delivery control method: The successful validation of the former two parts facilitated the drug delivery control system based on hollow microneedle patches through circuit design and 3D printing. In this part, the real-time blood glucose concentration can be determined by altering the capacitance and converting it into error signals for the automatic drug delivery system to achieve precise control.

#### 4.3.1. Preparation of the PVA-PEG-MoS_2_ Hydrogel

**Figure 13:**
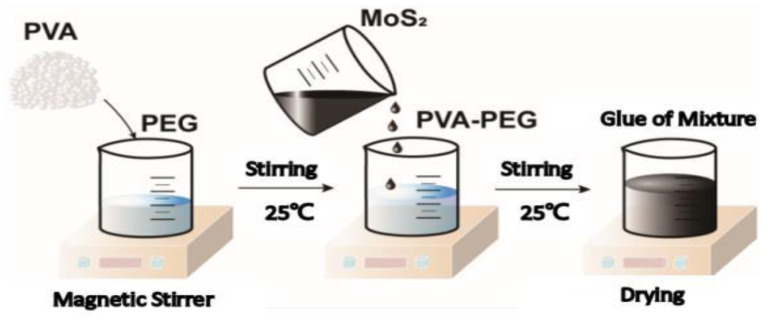
Optimized preparation process.

The results of previous research indicate that films with varying flexibility can be obtained by mixing the dispersion of MoS_2_ nanosheets with a self-made encapsulant, spin-coating with a spin-coater, and drying at room temperature or slightly higher than room temperature, based on different residual moisture levels [31,32]. With the drying process taking up 94.8% to 96.3% of the total preparation time and being prone to excessive water loss, our study optimized the preparation procedure in view of the preparation time. The PVA-to-PEG ratio was adjusted from 1:4 to 1:2 to increase the viscosity and reduce the drying time, and a pre-drying step was introduced to expand the application scenarios of the colloid (as illustrated in Figure 13).

(1) Preparation Results

The reagent quantities were recorded, as shown in Table 2. The MoS_2_ nanosheets were mixed and stirred with PVA-PEG at room temperature for 4 h. After a sufficient cross-linking reaction had taken place, the resulting mixture was divided into 6 mL portions, which were then placed in a draft-free thermostatic drying oven at 35 °C until the hydrogel formed.

#### 4.3.2. Improvement in Microfluidic Chip Design

**Figure 14:**
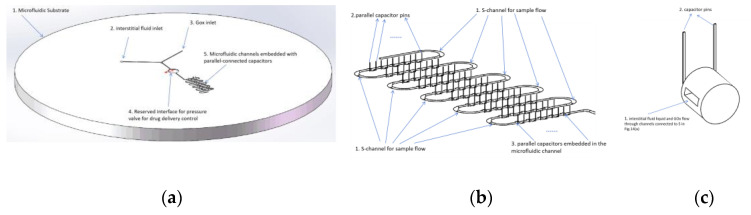
Microfluidic chip design: (**a**) annotation of liquid injection into the microfluidic chip; (**b**) microcapacitors embedded in the microfluidic channels; and (**c**) capacitive sensors based on PVA-PEG-MoS_2_ nanosheet hydrogels.

The inlets of the microfluidic chip indicate interstitial fluid samples (extracted using a microneedle pressure structure and injected at position 2 in Figure 14a) and glucose oxidase solution (GOx; injected from the storage tank at position 3 in Figure 14a synchronously), as depicted in Figure 14a. A set of capacitors was embedded within the microfluidic channels of the chip, ensuring that the mixed sample fluid flowed through each capacitor-reserved channel, as depicted in Figure 14b. The leads of this set of capacitors were connected in parallel and ultimately linked to the data acquisition pins of the chip, as illustrated in Figure 14c.

#### 4.3.3. Calibration of Microcapacitors to Glucose Concentration in Interstitial Fluid

- Capacitors connected in parallel.

1. Necessary definitions are provided as follows:

▪ Initial state: Defined as the state before the microfluidic chip is used. In this case, the total capacitance is specified in Equation (1):

(1) $$C_{total(0)}=\sum_{i=1}^{N}C_{i\max}$$

where $C_{total(0)}$ is defined as the total capacitance of the capacitors connected in parallel in the initial state (before their use).

The subscript (0) indicates the initial moment.

N=1,2,⋯ is the total number of capacitors connected in parallel.

i=1,2,⋯N is the ID of the capacitors connected in parallel.

▪ Depleted state: Defined as the state in which all MoS_2_ nanosheets embedded in the microfluidic chip are consumed or changed in property, which results in an ignored capacitance change. In this case, the total capacitance is specified in Equation (2):
  (2) $$C_{total(f)}=\sum_{i=1}^{N}C_{i\min}$$
  where $C_{total(f)}$ is the total capacitance of the capacitors connected in parallel in the depleted state.

The subscript (f) indicates the depleted state of each capacitor.

$C_{i\min}$ is the final capacitance when the i-th capacitor reaches depletion (after the consumption of MoS_2_ nanosheets, the capacitance no longer varies, or shows little variation, and this value is defined as the final value).

▪ Intermediate state: Defined as the state in which the capacitors embedded in the microfluidic chip start reacting with glucose in the interstitial fluid. In this case, the total capacitance is expressed in Equation (3):

(3) $$C_{total}(k)=\sum_{i=1}^{n}C_{i\max}+\sum_{i=n+1}^{N}C_{i\min}+C_{j}$$

k: The k-th sampling and delivery of interstitial fluid;

$C_{j}$: The capacitance value of the j-th capacitor currently undergoing reaction;

n: The number of capacitors in the initial state.

2. The relationship between the capacitance change and the signal period from the 555 chip is as follows:

① It is known that the pulse width of the periodic square-wave signal provided by the 555 chip is calculated as shown in Equation (4):

(4) $$t_{w}(k)=RC_{total}(k)\ln3$$

where

$t_{w}$: The pulse width of the 555 chip, in seconds (s);

R: The equivalent resistance of the 555 chip, a constant.

② The expression for the initial capacitance of the specific capacitor is determined using Equation (5):

(5) $$C_{\max}=\frac{\varepsilon_{0}\times S}{d}$$

where

$\varepsilon_{0}$: The relative dielectric constant measured upon fabrication of a capacitor;

S: The effective relative area between electrode plates, a constant;

d: The effective distance between electrode plates, a constant.

③ The expression for the depleted capacitance of the specific capacitor is determined using Equation (6):

(6) $$C_{\min}=\frac{\varepsilon_{0}-\Delta \varepsilon_{\max}}{d}S$$

where

$\Delta \varepsilon_{\max}$ is defined as the maximum change in dielectric constant when a specific capacitor is depleted;

$C_{\min}$, $C_{\max}$, and $\Delta \varepsilon_{\max}$ are treated as constants in specific calculations, as they are only associated with the mass fraction of MoS_2_ doped in the dielectric under ideal conditions where the capacitors reached complete depletion.

④ Based on the expansion of Equation (3), the relationship between the real-time total capacitance and changes in the dielectric constant during the catalytic reaction after multiple capacitors connected in parallel can be further obtained, as shown in Equation (7):

(7) $$C_{total}(k)=n_{k}C_{\max}+(N-n_{k}-1)C_{\min}+\frac{(\varepsilon_{0j}-\Delta \varepsilon_{j})S_{j}}{d_{j}}$$

where

$\varepsilon_{0j}$: The relative dielectric constant measured upon fabrication of the j-th capacitor;

$\Delta \varepsilon_{j}$: The maximum change in dielectric constant when the j-th capacitor is depleted;

$S_{j}$: The effective relative area between electrodes in the j-th capacitor, a constant;

$d_{j}$: The effective distance between electrodes in the j-th capacitor, a constant;

$n_{k}$: The number of capacitors that remained unused after the k-th test.

In Equation (7), $C_{\min}$, $C_{\max}$, and $\varepsilon_{0j}$ are only associated with the mass fraction of MoS_2_ nanosheets doped in the MoS_2_ hydrogel dielectric; N is fixed when the microfluidic chip is fabricated and it can be treated as a constant. In specific applications, the real-time unconsumed capacitance is known as a constant. Therefore, $n_{k}$ is also a constant, which can be obtained using the following method:

⑤ Judgment of the number of capacitors in the initial state.

Equations (1)–(3) yield the simultaneous Equation (8), which represents the total capacitance of the microfluidic chip when the j-th capacitor has not yet participated in the reaction but is about to, and when the j-th capacitor has just been depleted and before the next capacitor starts to deplete, respectively:

(8) $$\left\{\begin{array}{l} C_{j\min}\to {\left[C_{Total}(j)\right]}_{\min}=n_{k}C_{\max}+(N-n_{k})C_{\min}\\ C_{j\max}\to {\left[C_{Total}(j)\right]}_{\max}=(n_{k}+1)C_{\max}+(N-n_{k}-1)C_{\min} \end{array}\right.$$

Equation (8) illustrates that the consumption of each capacitor contributes to a fixed capacitance characteristic range for the total capacitance. Therefore, the capacitance change in the j-th capacitor being consumed can be represented using Equation (9), which characterizes its specific range.

(9) $${\left[C_{total}(j)\right]}_{\min}<C_{total}(j)<{\left[C_{total}(j)\right]}_{\max}$$

The range of each capacitor in the capacitors connected in parallel can be directly calibrated using Equation (9) to form a table, which can be referenced during subsequent use. As measured experimentally, when the mass fraction of the doped MoS_2_ is greater than 2%, the dielectric constant of the prepared hydrogel varies almost proportionally with the mass fraction:

(10) $$\Delta \varepsilon_{j}=K\times \Delta m\%$$

where

K is the proportionality coefficient between the MoS_2_ dielectric and mass fraction. Once the initial mass fraction m% is determined and greater than 2%, K becomes a fixed coefficient and is typically calibrated during manufacturing. K turns into 0 as MoS_2_ is consumed to below 2%.

Δm% indicates the MoS_2_ mass fraction consumed in real time.

During the catalytic process, MoS_2_ undergoes a specific chemical reaction, turns into molybdenum trioxide, and is precipitated out of the hydrogel. The mass fraction of MoS_2_ decreases equivalently during the catalytic reaction. Assuming that, after the k-th test, the blood glucose is accumulated to $g_{x}(k)$, then the real-time blood glucose concentration can be expressed as Equation (11):

(11) $$c_{x}(k)=\frac{g_{x}(k)-g_{x}(k-1)}{V}=\mu \times \Delta m\%$$

where

$g_{x}(k)$: The blood glucose consumed in the microfluidic chip in total after the k-th test;

$g_{x}(k-1)$: The blood glucose consumed in the microfluidic chip in total after the (k − 1)-th test;

$c_{x}(k)$: The real-time blood glucose concentration, in mmol/L;

*V*: The volume of interstitial fluid extracted each time, in L;

μ: The consumption coefficient during the catalytic process, in mmol^−1^, a constant at a given temperature.

By combining Equations (10) and (11) and eliminating Δm%, the relationship between the real-time change in the dielectric constant during catalysis and the real-time blood glucose concentration can be derived from Equation (12).

(12) $$\Delta \varepsilon =\frac{K}{\mu }c_{x}(k)V$$

By combining and organizing Equations (7), (11), and (12), the relationship between the real-time total capacitance during catalysis $C_{total}(k)$ and the change in capacitance at the time of calibration can be presented as Equation (13):

(13) $$\Delta \varepsilon =\varepsilon_{0}-\frac{d}{S}[C_{total}(k)-n_{k}C_{\max}-(N-n_{k}-1)C_{\min}]$$

After trimming, Equation (14) is obtained:

(14) $$C_{total}(k)=\frac{t_{w}(k)}{R\ln3}$$

By combining Equations (14) and (13) and transforming them, the relationship between the real-time blood glucose concentration and real-time total capacitance can be presented as Equation (15):

(15) $$g_{x}(k)=-\frac{\mu d}{KSR\ln3}t_{w}(k)+\frac{\mu \varepsilon_{0}}{K}+\frac{\mu d}{KS}[nC_{\max}+(N-n-1)C_{\min}]$$

As $g_{x}(k)$ is the blood glucose consumed from the 1st to the k-th test, the relationship between the real-time concentration of glucose, $c_{x}(k)$, and the pulse width change, $t_{w}(k)$, is presented as Equation (16).

(16) $$c_{x}(k)=\frac{g_{x}(k)-g_{x}(k-1)}{V}=-\frac{\mu d}{KVSR\ln3}[(t_{w}(k)-t_{w}(k-1)]+\frac{\mu d}{KVS}(n_{k}-n_{k-1})(C_{\max}-C_{\min})$$

- Capacitors connected in series

If the microcapacitors embedded in the microfluidic chip are connected in series, the expressions for the initial capacitance, depleted capacitance, and total capacitance, and the relationship between the real-time pulse width of the timer chip and real-time blood glucose concentration are as illustrated in Equations (17)–(20), respectively:

(17) $$\frac{1}{C_{total(0)}}=\sum_{i=1}^{N}\frac{1}{C_{i\max}}$$

(18) $$\frac{1}{C_{total(f)}}=\sum_{i=1}^{N}\frac{1}{C_{i\min}}$$

(19) $$\frac{1}{C_{total}(k)}=\sum_{i=1}^{n_{k}}\frac{1}{C_{i\max}}+\sum_{i=n+1}^{N}\frac{1}{C_{i\min}}+\frac{1}{C_{j}}$$

(20) $$c_{x}(k)=\frac{g_{x}(k)-g_{x}(k-1)}{V}=\frac{\mu \varepsilon_{0}}{KV}\frac{RD\ln3-\alpha_{2}(k)t_{w}(k)}{RD\ln3-\alpha_{1}(k)t_{w}(k)}-\frac{g_{x}(k-1)}{V}$$

where $\alpha_{1}(k)$, $\alpha_{2}$, and D are constants and can be represented by Equations (21), (22), and (23), respectively:

(21) $$\alpha_{1}(k)=n_{k}SC_{\min}+(N-n_{k}-1)S_{j}C_{\max}$$

(22) $$\alpha_{2}(k)=n_{k}S_{j}C_{\min}+(N-n_{k}-1)S_{j}C_{\max}+d_{j}C_{\max}C_{\min}$$

(23) $$D=S_{j}C_{\min}C_{\max}$$

#### 4.3.4. Circuit Design Based on the 555 Chip

**Figure 15:**
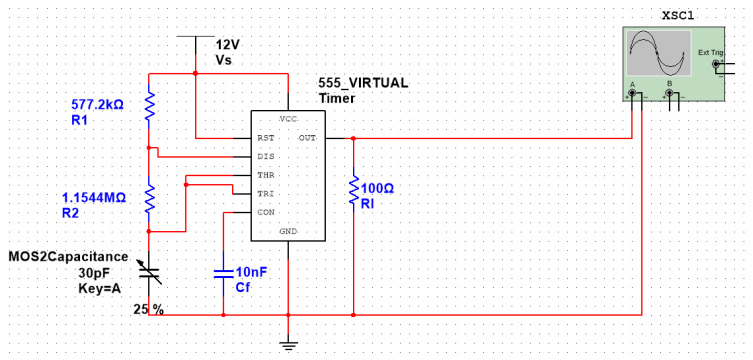
Design of the microfluidic chip capacitance–signal period conversion circuit based on the 555 chip.

The 555 timer chip was selected to provide square-wave inputs, and the discharge time of the capacitor can be measured by changing the capacitance, as shown in Figure 10. The 90% discharge time of the capacitor can be obtained through an MCU calculation to infer the capacitance change and blood glucose concentration. The circuit design is shown in Figure 15, and the total capacitance of parallel microcapacitors is presented as 30 pF. Therefore, according to Figure 10, with 30 pF of microcapacitors (100%), the output of the 555 chip is marked with green; with 15 pF of microcapacitors (50%), the output is marked with blue; and, finally, with 7.5 pF of microcapacitors (25%), the output is marked with yellow.

### 4.4. Drug Delivery Process

The MoS_2_ hydrogel capacitance sensor monitoring device can transform the detected real-time glucose concentration chemical signals into capacitance-modulated electrical signals, and further convert them into easily measurable timing signals. The MoS_2_ hydrogel capacitance sensor was connected to the central microelectronic chip. The central microelectronic chip analyzed and processed the electrical signals according to the preset program and algorithm.

The electromagnet injected insulin or other glucose-controlling drugs based on the electrical signals from the central microelectronic chip. The central microelectronic chip was connected to the electromagnet and Bluetooth transmission module of the drug delivery device. According to the processed electrical signals, the device determines whether to drive the electromagnet to inject insulin. If an injection is needed, the injection duration can be controlled according to the preset dosage of insulin or other glucose-controlling drugs, as shown in Equation (24):

(24) $$t_{c}=\beta (c_{x}-c_{0})$$

where

$t_{c}$: The control time to open the electromagnetic valve or other actuators;

$c_{0}$: The standard blood glucose, a constant;

β: The influence coefficient of the glucose-lowering drugs used on the blood glucose concentration, in s·Lmol.

The inlet of the microfluidic chip of the drug delivery device as connected to the liquid reservoir, while the outlet was designed to connect to the drug delivery microneedle patch. When the microperistaltic pump is driven by the signal from the blood glucose calibration, insulin from the reservoir is expected to be injected by the microfluidic chip via the drug delivery microneedle, thereby achieving drug delivery.

**Figure 16:**
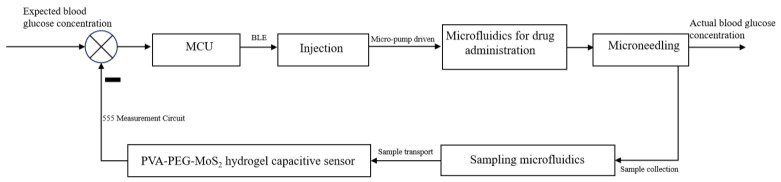
Block diagram of drug delivery control based on microneedles.

As shown in Figure 16, the MCU can calculate the remaining insulin or other glucose-controlling drugs based on the injected dosage. This is then transmitted to the client side in a timely manner via the Bluetooth transmission module. When the drug is about to be depleted, the device can provide a reminder signal to replenish insulin or other glucose-controlling drugs in the reservoir or replace the reservoir.

## 5. Conclusions

In this study, a novel blood glucose online monitoring method was proposed to provide signals for blood glucose control by converting the glucose concentration data into pulse width, making use of the sensitivity of low-doped PVA-PEG-MoS_2_ hydrogels to the glucose reaction and the output pulse width changing with the capacitance change in the 555 chip. Three significant modules were validated to determine the feasibility of the proposed method. (1) The feasibility of PVA-PEG-MoS_2_ hydrogels was measured. The results show that a 2% mass ratio of MoS_2_ nanosheets is the critical point of permittivity change. (2) The connection method of microcapacitors embedded in microfluid channels was validated using mathematical deduction and simulated in MATLAB. The results show that the parallel connection method provides excellent linearization, which proved to be more appropriate as a sensor. (3) Finally, it was determined that the pulse width change in the 555 chip output varies with the total capacitance change, and the circuit was designed and validated using Multisim. Therefore, the proposed novel method for continuous online monitoring is effective and, in accordance with microneedle technology, this method can improve the online monitoring compliance of patients with diabetes.

## Figures and Tables

**Table 1:** Dielectric constant and dielectric loss results.

Group	Measurement Frequency (Hz)	Permittivity	Dielectric Loss (S/CM)
Group 1	1000	53.7711	1.49991
Group 2	1000	48.5774	2.03166
Group 3	1000	158.951	9.28279
Control Group	1000	28.1465	0.549981

**Table 2:** Records of experimental reagent quantities.

Group	MoS_2_ Nanosheets *	PVA-PEG Embedded Media	Distilled Water
Group 1 (0.8%)	40 mg	5.000 g	60 mL
Group 2 (1.2%)	60 mg	5.000 g	40 mL
Group 3 (2%)	100 mg	5.000 g	0 mL
Control Group (0%)	0 mg	5.000 g	100 mL

* Specifications of the dispersion of MoS_2_ nanosheets: 1 mg/1 mL. Here, 40 mL, 60 mL, and 100 mL are used.

## Data Availability

The original contributions presented in the study are included in the article, further inquiries can be directed to the corresponding author/s.
